# Sex differences in sympathetic gene expression and cardiac neurochemistry in Wistar Kyoto rats

**DOI:** 10.1371/journal.pone.0218133

**Published:** 2019-06-13

**Authors:** Richard G. Bayles, Joanne Tran, Antoinette Olivas, William R. Woodward, Suzanne S. Fei, Lina Gao, Beth A. Habecker

**Affiliations:** 1 Department of Physiology and Pharmacology, Oregon Health & Science University, Portland, Oregon, United States of America; 2 Bioinformatics & Biostatistics Core, Oregon National Primate Research Center, Oregon Health & Science University, Beaverton, Oregon, United States of America; Hudson Institute, AUSTRALIA

## Abstract

The stellate ganglia are the predominant source of sympathetic innervation to the heart. Remodeling of sympathetic nerves projecting to the heart has been observed in several cardiovascular diseases, and sympathetic dysfunction contributes to cardiac pathology. Wistar Kyoto rats are a common model for the study of cardiovascular diseases, but we lack a profile of the baseline transcriptomic and neurochemical characteristics of their cardiac sympathetic neurons. Most studies of cardiovascular disease have used male animals only, but in the future both male and female animals will be used for these types of studies; therefore, we sought to characterize the transcriptome of male and female stellate ganglia and to correlate that with catecholamine and acetylcholine content in the heart. We have generated a dataset of baseline RNA expression in male and female Wistar Kyoto rat stellate ganglia using RNA-seq, and have measured neurotransmitter levels in heart and stellate ganglia using HPLC and mass spectrometry. We identified numerous gene expression differences between male and female stellates, including genes encoding important developmental factors, receptors and neuropeptides. Female hearts had significantly higher neurotransmitter content than male hearts; however, no significant differences were detected in expression of the genes encoding neurotransmitter synthetic enzymes. Similarly, no statistically significant differences were identified between the sexes in cardiac tyrosine hydroxylase levels.

## Introduction

As researchers increasingly recognize the importance of studying both male and female animals in disease models, it becomes increasingly clear that a sound understanding of the baseline physiology needs to be established in both sexes, in line with ongoing efforts of the NIH [1]. Stellate ganglia contain the majority of sympathetic neurons projecting to the heart, and we previously generated a reference transcriptome from mouse stellate ganglia with associated tissue neurochemistry [2]. Significant differences were detected between the sexes both in terms of gene expression and atrial neurotransmitter levels, highlighting the need to identify this baseline information in both sexes.

Rats, like mice, are a prominent model organism used in studies of cardiovascular diseases. Most studies, however, use only male rats to investigate the mechanisms that underlie pathology. This is true for studies of a broad range of diseases including heart failure, arrhythmias, and hypertension that utilize a variety of different rat strains [3–7]. Recently, Bardsley and colleagues compared the stellate ganglion transcriptome of Wistar rats with spontaneously hypertensive rats (SHR)[8, 9]. Differences were observed in the expression of genes related to calcium handling and cyclic nucleotide signaling that were consistent with alterations in neuronal calcium handling and cyclic nucleotide metabolism identified in physiological experiments [7, 10]. This type of work underscores the promise of transcriptomics to identify underlying mechanisms of disease and potential sites for intervention. However, these transcriptomic studies—like most other studies of cardiovascular disease—utilized only male rats.

Our study aims to provide baseline transcriptome and neurochemistry data on male and female adult Wistar Kyoto (WKY) rats, which are a common strain used in cardiovascular studies. RNA-seq analysis of stellate ganglia identified numerous sex-based differences in gene expression and several of these were confirmed by real-time PCR. Sex differences were also identified in cardiac neurotransmitter levels, with higher levels of both norepinephrine (NE) and acetylcholine (ACh) identified in female atria and elevated NE found throughout the female ventricle. Comparison with our previous mouse data suggests limited overlap between the two species in genes that exhibit sex-based differences in expression.

## Materials and methods

### Animals

This study was carried out in strict accordance with the recommendations in the Guide for the Care and Use of Laboratory Animals published by the National Academies Press (Ed 8). All procedures were approved by the Oregon Health & Science University Institutional Animal Care and Use Committee (protocol number IP00001366). Male and female Wistar Kyoto (WKY) rats (age matched 16–18 weeks old) were obtained from Jackson Laboratories, and maintained on a 12 h: 12 h light/dark cycle with *ad libitum* access to food and water. Male rats were consistently heavier than female rats (Mean ± SEM: 198.3 ± 2.6 g for females and 317 ± 8.9 g for males, p<0.001). Rats were sedated with isoflurane, and the heart and sympathetic ganglia were rapidly dissected. Left and right atria and ventricles were separated, as were the left and right stellate and superior cervical ganglia (SCG). The left ventricles were further separated into apex, mid, and base sections. Tissues collected for RNA were placed in RNAlater (Qiagen) and stored at -20°C. Tissues from a second set of animals collected for neurochemical analyses were snap frozen and stored at -80°C.

### RNA-seq

RNA was extracted from ganglia using the RNAqueous Micro kit (Ambion) according to the manufacturer’s instructions as previously described[2]. The DNAse treatment was performed, and samples were stored at -80°C. Each sample was assessed for RNA quality and integrity using a 2100 Bioanalyzer Instrument (Agilent). Samples with low quality RNA (RIN <6) were not included. Sequencing was performed using the right stellate ganglion from 5 males and 7 females (RIN range 7.1–9.7, average 8.6 with no difference between sexes). Sequencing libraries were prepared using the TruSeq Stranded Total RNA Library Prep Kit with Poly A enrichment (Illumina) using 130 ng of RNA per sample. Paired-end sequencing (100 bp reads, 2 lanes of 6 samples per lane, sexes evenly randomized) was performed using an Illumina HiSeq 2500 through the Massively Parallel Sequencing Shared Resource at OHSU.

### Bioinformatics

The quality of the raw sequencing files were evaluated using FastQC[11] combined with MultiQC[12] (http://multiqc.info/). The files were imported into ONPRC’s DISCVR-Seq[13], LabKey[14] Server-based system, PRIMe-Seq. Trimmomatic[15] was used to remove any remaining Illumina adapters. Reads were aligned to the Rattus_norvegicus.Rnor_6 genome in Ensembl along with its corresponding annotation, release 92. The program STAR (v020201)[16] was used to align the reads to the genome. STAR has been shown to perform well compared to other RNA-seq aligners [17]. Two-pass mode was used. The program featureCounts (v1.6.0) [18] was used to count the number of strand-specific reads that aligned to each gene. Multi-mapping reads were fractionally distributed to each potential gene source. RNA-SeQC (v1.1.8.1) [19] was utilized to ensure alignments were of sufficient quality. Raw RNA-seq data consisted of an average of 74M reads per sample with 94% mapping to the reference genome. All samples had similar mapping rates and other quality control (QC) metrics—none were determined to be outliers based on QC.

Gene-level raw counts were filtered to remove genes with extremely low counts in many samples following the published guidelines[20]. Specifically, genes with extremely low counts were defined as those having no more than 0.3 counts per million (CPM) in a sample, corresponding to a count of 7–8 in the smallest library. Genes passing extreme low count criteria in at least 5 samples were kept, ensuring the retention of genes only expressed in the smallest group. 15977 genes were retained after extreme low count filtering.

Gene-level differential expression analysis was performed in open source software R[21]. Gene-level raw counts were normalized using the trimmed mean of M-values method (TMM)[22] and transformed to log-counts per million with associated observational precision weights using the voom[23] method. Gene-wise linear models comparing the sexes were employed for differential expression analyses using limma with empirical Bayes moderation[24] with false discovery rate (FDR) adjustment[25].

### Real-time PCR

Select expression differences were confirmed using additional RNA from same right stellate ganglia that were sequenced. Reverse transcription was performed using the iScript reverse transcription supermix (BioRad) according to the manufacturer’s instructions. The following TaqMan Gene Expression Assays (Thermo-Fisher) were used for real-time PCR analysis: *Gapdh* endogenous control, Rn99999916_s1; *Th*, Rn00562500_m1; *Ret*, Rn01463098_m1; *Hpca*, Rn04222958_g1; *Penk*, Rn00567566_m1; *Nr4a2*, Rn00570936_m1; *Htr2b*, Rn00691836_m1; *Htr3b*, Rn00573408_m1; *Egr1*, Rn00561138_m1.

### HPLC analysis of Norepinephrine (NE) and mass spectrometry analysis of Acetylcholine (ACh)

Frozen tissue was used for neurotransmitter analysis as described previously[26]. All tissue was homogenized in perchloric acid (300 μl, 0.1 M) containing dihydroxybenzylamine (1.0 μM) as an internal standard. Ganglia were homogenized without weighing. Atria were weighed and added directly to the homogenization buffer. Right ventricles and sections of the left ventricle were pulverized using a mortar and pestle on dry ice, prior to weighing and homogenization of a small amount of tissue. After homogenization, all samples were centrifuged (13,000 g for 5 min). Catecholamines were purified from an aliquot (100 μL) of the supernatant by alumina adsorption. A second aliquot (100 μL) was filtered at 4°C for ACh quantification. NE, the NE metabolite dihydroxyphenylglycol (DHPG), dopamine (DA), and the dopamine metabolite 3,4-dihydroxyphenylacetic acid (DOPAC) were measured by HPLC with electrochemical detection, and ACh was quantified by mass spectrometry as described previously[26].

### Statistics

Graphing and statistical analyses were performed using Microsoft Excel and GraphPad Prism v6. Binary comparisons between males and females for individual genes (qPCR) were performed using Student’s t-tests. Comparison of two data sets with unequal variances used an unpaired t-test with Welch’s correction. Neurotransmitter levels across different parts of the same hearts were compared using two-way ANOVA with repeated measures, with p values adjusted for multiple comparisons.

## Results

**Fig 1:**
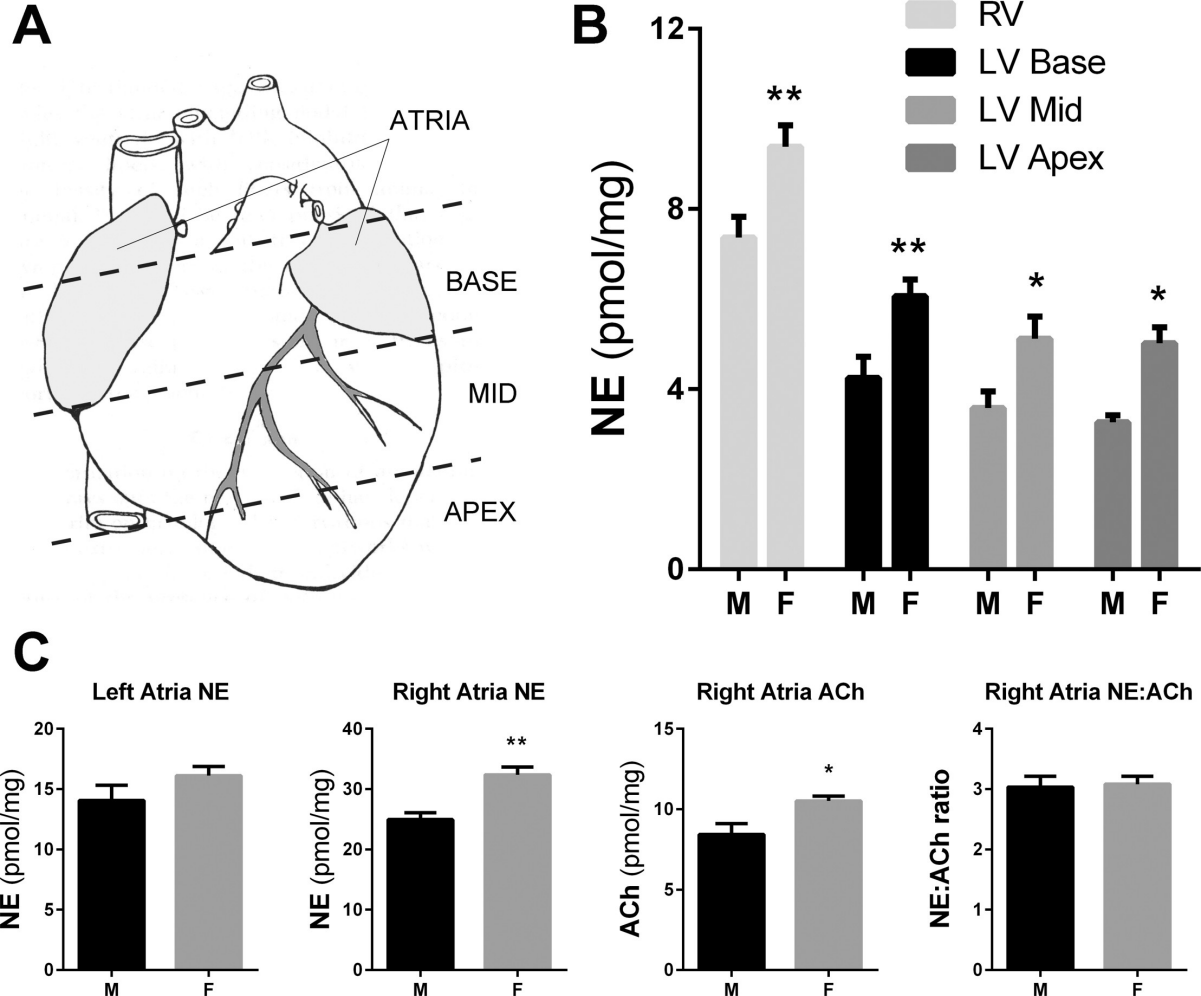
Female WKY rat hearts have higher neurotransmitter content than male hearts. A) Tissue collection schematic. Atria (shaded) were separated from the ventricles. The ventricles were cut into 4–5 mm sections highlighted by the dashed lines. Adapted from [28]. B) NE concentrations were higher in females than males in all areas of both the left and right ventricle. Mean±SEM, n = 6, *p<0.05, **p<0.01 female vs. male, 2-way ANOVA. Difference between regions of the ventricle, p<0.0001. C) Both NE and ACh were higher in the right atria of female rats compared to males, while the ratio of NE to ACh did not change. Mean±SEM, n = 6; (except Female RA n = 5) *p<0.05, **p<0.01 female vs male, unpaired t-test.

To characterize baseline neurochemical characteristics of adult WKY rat hearts, norepinephrine (NE) and acetylcholine (ACh) were measured in atria, the right ventricle, and the base, mid-wall and apex of the left ventricle. A base to apex gradient in NE concentration is a well-established feature of cardiac physiology [27] and our data confirmed this gradient for WKY rats (region-specific differences p<0.0001) (**Fig 1**). In addition, a higher concentration of NE was observed across all regions of the heart in females compared to males. In right atria, a higher concentration of ACh was also observed in females compared to males, proportional to the increase in NE. Surprisingly, the NE metabolite DHPG was not detectable in any of the rat heart tissue samples, although the DHPG standards were readily detected.

Catecholamines and their metabolites were also quantified in left and right stellate ganglia. Both NE and dopamine were detected in rat stellates, with similar levels in male and female ganglia (**Table 1**). The dopamine metabolite DOPAC was significantly higher in left female stellate ganglia compared to males, and female left stellates also had a significantly lower NE to dopamine ratio than male left stellates. Male and female right stellate ganglia exhibited similar levels of all detected catecholamines and their metabolites, in contrast to the sex differences identified in cardiac catecholamine content.

**Table 1:** Neurotransmitter and metabolite levels in the stellate ganglia.

Stellate ganglia		NE (pmol/mg)	DA (pmol/mg)	DOPAC (pmol/mg)	NE : DA ratio
**LEFT**	Female Male	185.9 ±31.8204.8 ±21.4	10.9 ±1.815.6 ±1.7	10.4 ±1.515.2 ±1.1*	17.1 ±0.713.3 ±0.6 **
**RIGHT**	Female Male	232.2 ±7.9210.9 ±10.4	20.1 ±2.617.5 ±0.5	17.3 ±4.311.4 ±0.7	12.4 ±1.312.2 ±0.9

Norepinephrine (NE) and Dopamine (DA) concentrations were not different between the sexes, and the NE metabolite DHPG was not detected. The DA metabolite DOPAC was significantly higher in the female left atria, and the NE : DA ratio was lower in the female left atria. Mean ±SEM, n = 6 *p<0.05, **p<0.01 female vs male, Student’s t-test.

**Fig 2:**
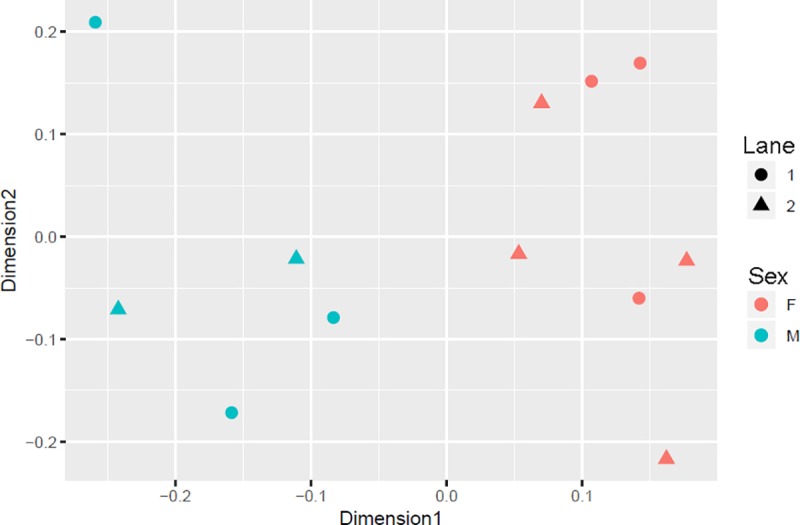
Multidimensional scaling plot of log-CPM values. MDS plot over 2 dimensions highlighting clear separation of sexes. F, Female Stellate (Red); M, Male Stellate (Blue). Samples were sequenced across 2 lanes (circles and squares). The distances on the plot approximate the typical log2 fold changes (Normalised CPM, counts per million) between the samples.

To identify transcriptomic differences between male and female stellate ganglia, and determine if those contributed to the differences in cardiac neurotransmitter content, RNA-seq was performed using RNA from adult right stellate ganglia. Separation of the sexes was observed during multidimensional scaling (MDS) analysis to determine how the expression profiles of each sample compared to all others (**Fig 2**). Genes associated with sympathetic neurotransmission were among the most highly expressed genes, consistent with our previous studies in mice[2] and with previous analyses of male rat stellates[8, 9]. Similar to mice, the top 100 most highly expressed genes were almost identical in males and females (**S1 Table**). No significant differences were detected in the expression of catecholaminergic genes such as *Th* (tyrosine hydroxylase), *Dbh* (dopamine beta hydroxylase), *Slc18a2* (vesicular monoamine transporter 2), or *Slc6a2* (norepinephrine transporter), or other genes associated with neurotransmission (**S1–S8 Figs, S2 Table**). Differential expression analysis identified 22 genes more highly expressed in males, and 20 genes more highly expressed in females with FDR-adjusted p < 0.05, but the scale of fold difference between the sexes was small for some of the genes, particularly the ones on autosomes (**Table 2**). Differentially expressed genes were dispersed across many gene families, with no significant pathway enrichment observed after Panther v13[29] and DAVID gene ontology analysis[30].

**Table 2:** RNA-seq differential expression analysis in the stellate ganglion (Male vs Female).

Gene ID	Chromosome	Log2FC	p-value	FDR adjusted p-value
*Eif2s3y*	Y	11.28	5.5E-14	4.4E-10
*Kdm5d*	Y	9.84	4.0E-14	4.4E-10
*AC239701*.*4*	Y	5.54	2.2E-13	1.2E-09
*AC241873*.*1*	Y	8.44	4.6E-13	1.9E-09
*AC239817*.*3*	Y	4.36	9.0E-13	2.9E-09
*Ddx3*	Y	11.24	1.3E-12	3.3E-09
*RF02266*	X	-7.98	1.2E-10	2.7E-07
*AC239701*.*1*	Y	6.40	1.9E-10	3.4E-07
*Eif2s3*	X	-0.56	1.8E-10	3.4E-07
*Kdm6a*	X	-0.96	1.2E-09	2.0E-06
*AABR07039356*.*2*	X	-9.40	2.6E-08	3.8E-05
*Ddx3x*	X	-0.39	6.5E-07	8.7E-04
*Cpne9*	4	-0.56	1.4E-06	1.7E-03
*B3gat1*	8	-0.58	1.5E-06	1.8E-03
***Penk***	5	0.96	4.4E-06	4.7E-03
*Kdm5c*	X	-0.56	6.7E-06	6.7E-03
*Tfrc*	11	0.43	1.1E-05	9.7E-03
*Pkp1*	13	-0.91	1.0E-05	9.7E-03
*Pbdc1*	X	-0.35	1.5E-05	1.2E-02
*Rgs20*	5	0.46	1.5E-05	1.2E-02
*LOC102553010*	17	0.40	1.5E-05	1.2E-02
*Sqor*	3	0.27	2.1E-05	1.5E-02
*AABR07019083*.*1*	15	-0.59	2.5E-05	1.7E-02
*Angptl4*	7	-0.62	2.7E-05	1.7E-02
*Zfp36l1*	6	-0.41	2.6E-05	1.7E-02
*Tdh*	15	0.49	3.3E-05	2.0E-02
*Arid5b*	20	-0.66	3.6E-05	2.1E-02
*Prps2*	X	0.26	4.6E-05	2.6E-02
*Tspan1*	5	-0.79	5.1E-05	2.6E-02
*RGD1561849*	5	0.39	5.0E-05	2.6E-02
*Serpinb1b*	17	0.37	4.9E-05	2.6E-02
***Nr4a2***	3	-0.76	5.3E-05	2.6E-02
*Adgra2*	16	-0.37	5.9E-05	2.9E-02
***Htr3b***	8	0.49	6.5E-05	2.9E-02
*Fzd1*	4	-0.40	6.5E-05	2.9E-02
*Tmem151b*	9	-0.27	6.5E-05	2.9E-02
*Zcchc12*	X	0.27	7.6E-05	3.3E-02
*Adgrf4*	9	1.01	9.1E-05	3.8E-02
*Dnah6*	4	0.36	9.8E-05	4.0E-02
*Brinp1*	5	0.52	1.0E-04	4.1E-02
*Slc6a11*	4	-0.58	1.1E-04	4.5E-02
*Nt5dc2*	16	0.34	1.3E-04	4.9E-02

Genes that are the most differentially expressed between males and females, sorted by FDRp-value (False discovery rate adjusted p-value). log2FC is the fold difference in expression between males and females, where log2FC of 1 corresponds to fold difference of 2 (up-regulated) vs. female and log2FC of -1 corresponds to fold difference of 0.5 (down-regulated) vs. female. P-value is the uncorrected p-value comparing males and females. The most highly differentially expressed genes were located on the sex chromosomes, however autosomal genes were also significantly different between the sexes. **Bold**; Genes of interest confirmed by qPCR.

We previously carried out RNA-seq comparing male and female mouse stellate ganglia[2], and therefore assessed potential overlap of differentially expressed genes between rat and mouse. There were 12,289 genes in common between the rat and mouse analysis. Genes were excluded that didn’t have counterparts with the same gene name in the other species, or that were filtered out due to low counts. Surprisingly, very few genes exhibited similar sex-specific expression in stellate ganglia from both rat and mouse **(Table 3)**.

**Table 3:** Genes differentially expressed between the sexes in both rat and mouse stellate ganglia.

	M v F mouse		M v F rat	
Gene Name	log2FC	FDR adjusted p value	log2FC	FDR adjusted p value
*eif2s3y*	11.62	2.3E-10	11.28	4.4E-10
*kdm5d*	10.61	3.1E-09	9.84	4.4E-10
*kdm6a*	-0.83	5.1E-06	-0.96	2.0E-06
*kdm5c*	-0.52	8.6E-04	-0.56	6.7E-03

Genes that are differentially expressed between males and females in stellate ganglia from both rat and mouse. FDRp-value, false discovery rate adjusted p-value; log2FC, fold difference in expression between males and females.

**Fig 3:**
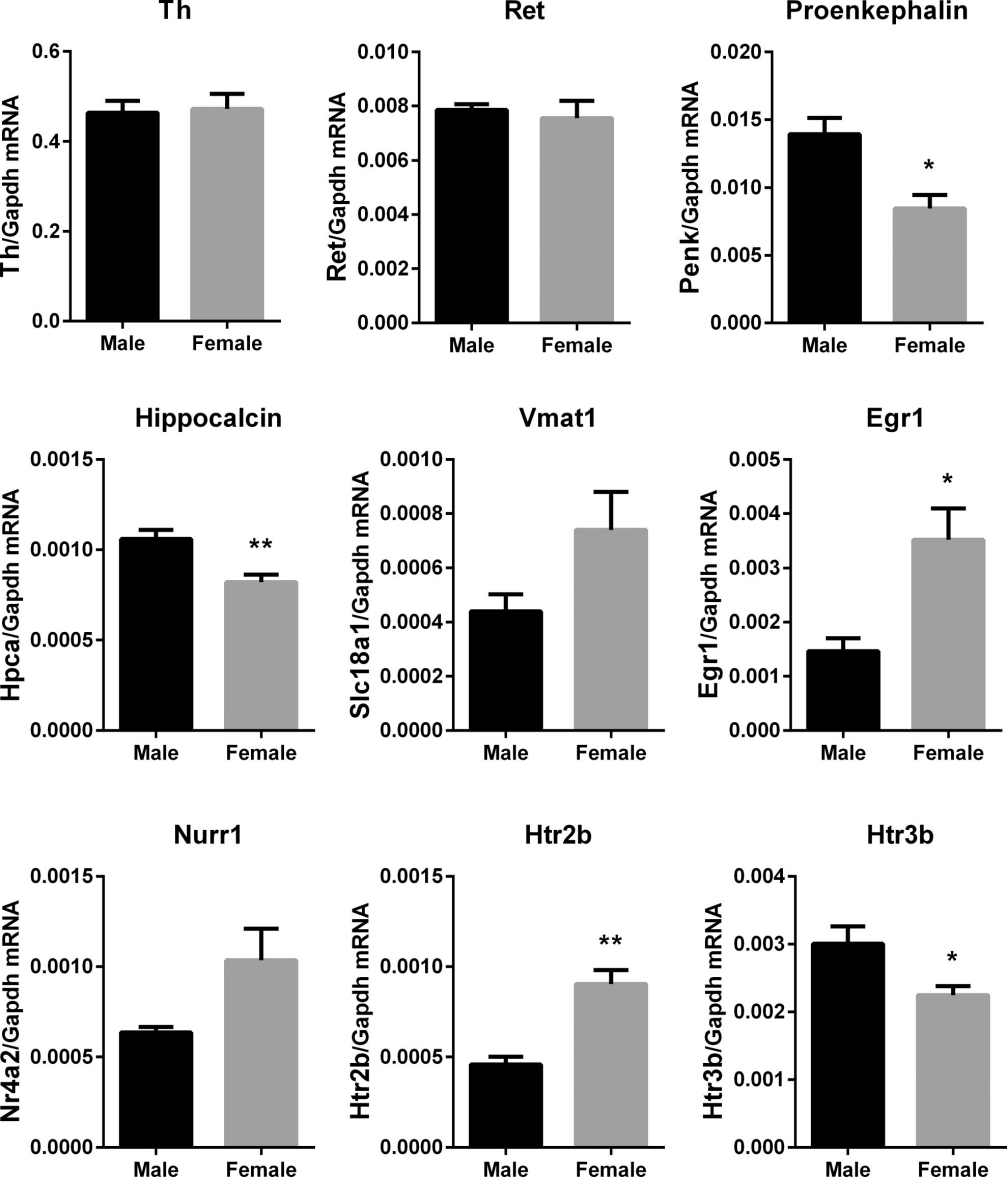
qPCR quantification of selected genes of interest in the stellate ganglion. Relative gene expression levels expressed as a ratio to *gapdh* expression in right stellate ganglion (Mean ± SEM, n = 6M vs 6F). There was strong agreement between RNA-seq and qPCR results for select genes of interest. *p<0.05, **p<0.01, ***p<0.001.

The non-X/Y genes that were different between the sexes in WKY rats included genes encoding developmental growth factors and serotonin receptors. A subset of genes that were in the top 50 most differentially expressed (**Table 2**) were selected for confirmation by qPCR, including *Penk* (Proenkephalin, the precursor polypeptide that generates the enkaphalins), *Nr4a2* (aka Nurr1, a transcription factor important in the development and maintenance of dopamine production), *Hpca* (Hippocalcin, a neuronal calcium sensor), *Egr1* (Early growth response protein 1, a developmental transcription factor), and *Htr2b* and *Htr3b* (Serotonin receptors). *Th* mRNA was assayed by qPCR as a gene that was not different between the sexes in mouse or rat, and *Ret* mRNA was quantified as a gene that exhibited differential expression in mouse stellates but similar expression in male and female rat stellates. PCR confirmed significantly higher expression of *Penk*, *Hpca* and *Htr3b* in the stellate ganglia of male rats, and significantly higher expression of *Egr1* and *Htr2b* in the stellate ganglia of female rats (**Fig 3**). *Th* and *Ret* mRNA were similar in both sexes confirming RNA-seq results.

**Fig 4:**
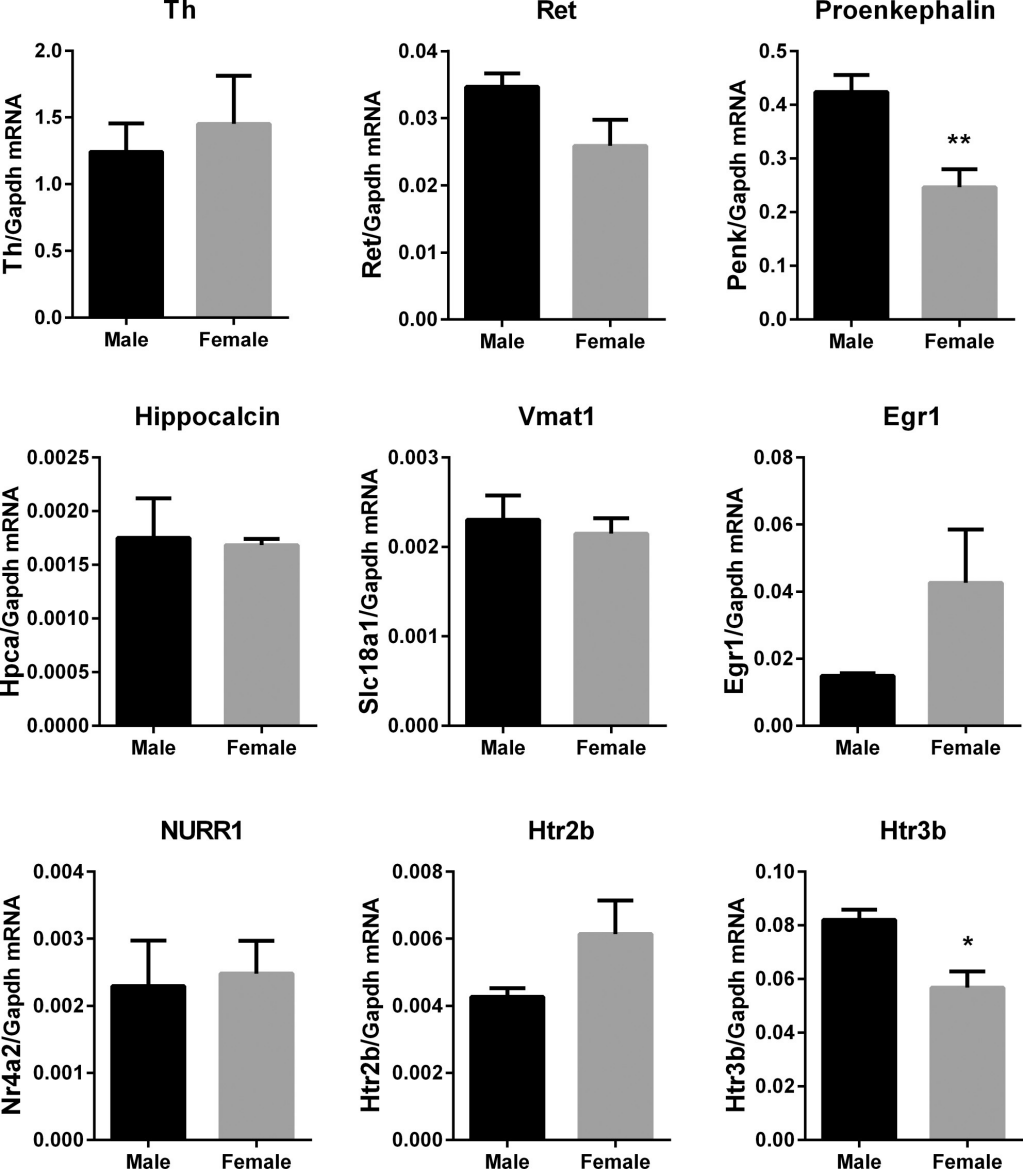
qPCR quantification of selected genes of interest in the SCG. Gene expression was assessed in the SCG as an alternative sympathetic ganglion. Relative gene expression levels expressed as a ratio to *gapdh* expression in right SCG (Mean ± SEM, n = 6M vs 6F). The gene expression levels of most genes were much higher relative to *gapdh* in the SCG compared to the stellate. The sex differences for *Penk* and *Htr3b* only were present in both the stellate and SCG. *p<0.05, **p<0.01, ***p<0.001.

To determine if the sex differences in gene expression observed in **Fig 3** were unique to stellate ganglia, the same genes were quantified by qPCR in another sympathetic ganglion, the superior cervical ganglion (SCG), from the same rats (**Fig 4**). The expression pattern of decreased *Penk* and *Htr3b* in female rats compared to males was conserved in stellate and the SCG, but there was no significant difference between the sexes in the expression of *Hpca*, *Egr1*, or *Htr2b* in the SCG.

**Fig 5:**
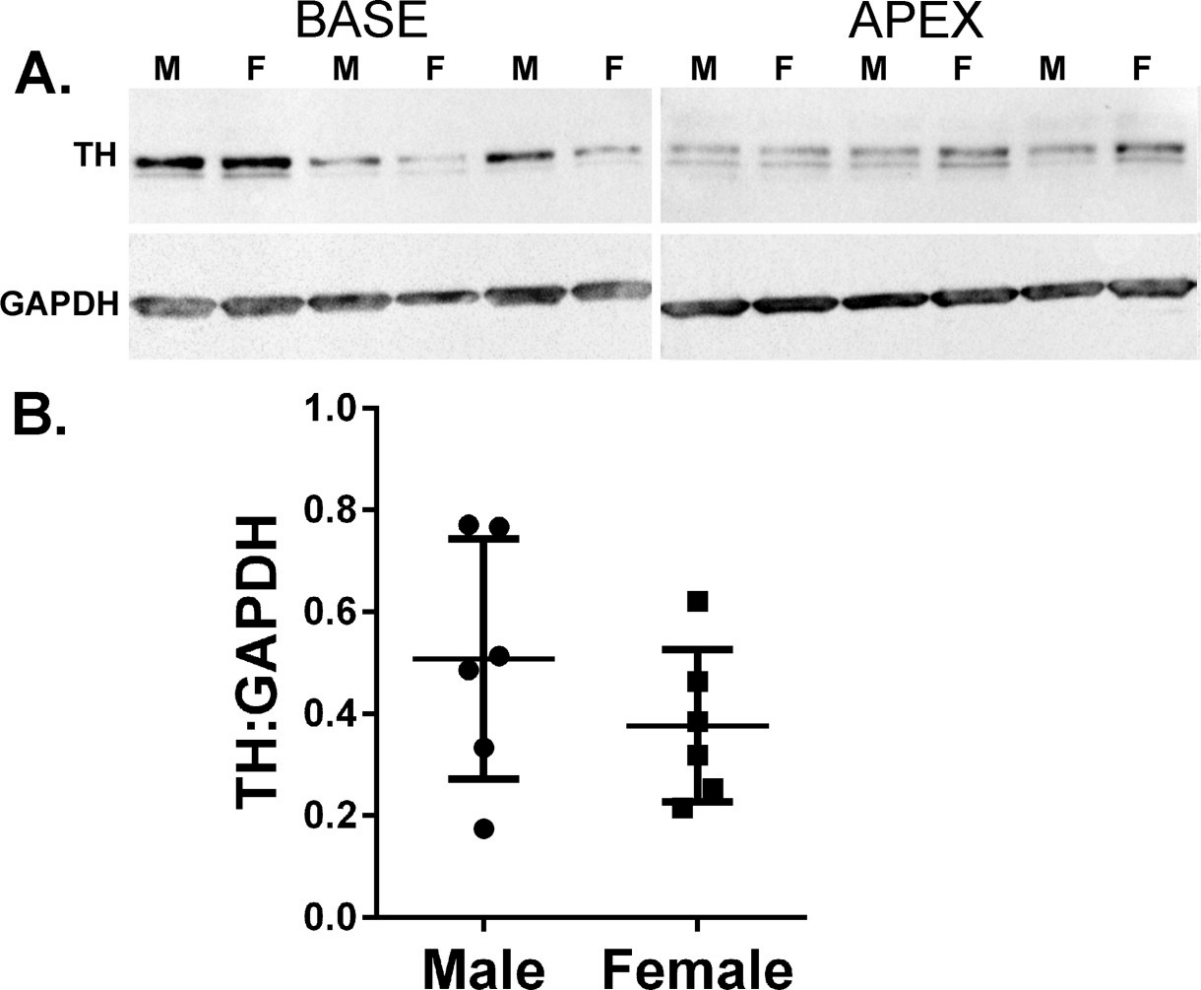
Tyrosine hydroxylase protein in left ventricle. (A) TH and GAPDH proteins were identified in the base (left) and apex (right) of the left ventricle by western blot and quantified via densitometry. Samples shown are base and apex from the same hearts. TH was normalized to GAPDH in the same sample. No significant differences were identified in either base or apex. (B) Average TH:GAPDH values for each heart (Mean ± SEM, n = 6/group).

Given the increased concentration of NE content in female hearts, and the lack of any differences in noradrenergic gene expression, we quantified TH protein in the left ventricle by western blot, assessing TH in the base and the apex (S9 and S10 Figs). TH levels in each region were highly variable between animals, even when normalized to GAPDH (Glyceraldehyde 3-phosphate dehydrogenase). There were no statistical differences in the TH levels of males compared to females in either the base or the apex of the left ventricle, nor was there a difference between sexes in the average TH content from each heart **(Fig 5)**.

## Discussion

In the present study we generated a reference transcriptome of stellate ganglia in male and female adult WKY rats with associated tissue neurochemistry. Statistically significant differences were detected between the sexes both in terms of gene expression and cardiac neurotransmitter levels. The most abundant genes within all ganglia related to neurotransmission, and these genes were present at identical levels in male and female ganglia. Genes that were differentially expressed between the sexes in rat stellate ganglia were distinct from those that were differentially expressed in male and female mouse stellates. Most of the genes that were differentially expressed in stellates were expressed at similar levels in male and female SCG, suggesting sex differences that were specific to the stellate. Neurotransmitter levels were similar in male and female stellate ganglia, but NE levels were significantly higher across all regions of female hearts compared to male hearts. No physiological parameters were quantified in this study, which focused on transcriptomics and neurochemistry.

There is a gradient of NE content and sympathetic nerve density from the base to the apex of the heart that is conserved across species [27, 31–33]. Our results confirmed the presence of this gradient across the rat heart, and for the first time identified significantly higher NE throughout the heart in female rats compared to male rats. This was unexpected, since we did not see a similar sex differential in cardiac NE levels in our earlier mouse study[2]. In order to understand if this sex difference in neurotransmitter content was common across rat strains, we re-examined NE data from previous studies carried out in Sprague Dawley rats, separating male (n = 6) and female (n = 4) animals in the unoperated control group[34]. We did not observe significant male-female differences in cardiac NE content (LV Mid-wall, M 5.8±0.7 vs F 6.8±0.4 pmol/mg, p = 0.3; RV, M 8.7±0.9 vs F 11.5±1.9 pmol/mg, p = 0.2)[34], consistent with identical cardiac physiology observed in the male and female rats[34]. We also compared our data to earlier studies that measured cardiac NE content in female Wistar rats [35] and male WKY rats [36]. Female Wistar rats had 6.9±0.8 pmol/mg NE in the RV and 3.3±0.3 pmol/mg in the intraventricular septum, while male WKY rats of a similar age had “myocardial” (presumably left ventricle) NE content of 4.8±0.4 pmol/mg. Our data are consistent with the general levels of NE previously quantified in WKY and Wistar hearts, but the differences in tissue collection and regional variation in NE content make it difficult to draw any conclusions related to sex-specific differences.

It is unclear what factors contributed to the higher NE content in female hearts, especially since NE content was identical in male and female stellate ganglia, which contain most of the sympathetic neurons projecting to the heart[37]. The male rats were significantly larger than the females of the same age, but NE concentrations were normalized to tissue weight, so differences in tissue size cannot explain the differential. We did not quantify nerve density in this study, but previous studies of nerve density did not identify any sex-based differences in the mouse heart [38, 39]. The rate limiting enzyme in NE synthesis is TH, and several studies have shown that the Y chromosome-linked, male-determining gene Sry regulates expression of the TH gene [40, 41] as well as genes encoding other enzymes required for NE synthesis [42]. Increasing expression of Sry *in vitro* or *in vivo* enhances expression of TH and other synthetic enzymes, which can lead to higher catecholamine content and blood pressure in male animals [42, 43]. In contrast to these previous studies, we found that TH gene expression was similar between the sexes in both stellate ganglia and SCG. Similarly, expression of all of the genes involved in synthesis, storage, release, reuptake and metabolism of NE were identical in male and female stellates (**S1–S8 Figs**). Although expression of the VMAT1 gene trended toward an increase in female stellate, it’s unlikely that this contributed to the difference in cardiac NE since its expression was near the limit of detection (3.5 CPM) whereas VMAT2, the major neuronal vesicular monoamine transporter, was expressed at similarly high levels (1600 CPM) in male and female ganglia.

Since NE content differed in the heart but not ganglia, we quantified TH protein in the left ventricle to determine if local changes in TH content could account for the differences in NE levels. TH levels were variable between animals of both sexes and no significant differences were observed between the sexes. We attempted to quantify TH activity using a colorimetric assay developed by Vermeer and colleagues [44], but it was not sensitive enough to detect TH activity in cardiac tissue extracts. TH activity is regulated by phosphorylation in sympathetic neurons, but we did not examine TH phosphorylation as a proxy for changes in enzyme activity because it does not consistently indicate an increase in enzyme activity. For example, phosphorylation at Ser40 can alleviate feedback inhibition of TH by catecholamines [45] but it can also trigger ubiquitination and degradation of the enzyme [46]. Enhanced reuptake of NE into nerve endings could also explain increased neuronal NE stores in female hearts. The DHPG:NE ratio can provide information on the efficiency of NE recycling into the pre-synaptic terminal, but we did not detect the NE metabolite DHPG in any of the rat tissue tested. This was surprising, as we and others have previously detected DHPG in mouse and rat heart [2, 35, 36] using the same or similar methods. However, careful analysis of chromatograms from our samples did not reveal a peak eluting at or near 3.2 minutes, when DHPG standards were detected, suggesting that the level of DHPG present after tissue processing was simply too low for detection. Thus, it remains unclear why NE content is consistently higher throughout the female heart in WKY rats, or if this contributes to desensitization of beta adrenergic receptor signaling on cardiac myocytes [47].

The genes that exhibited the greatest differential expression between sexes were those associated with the sex chromosomes, as expected. Other genes that were significantly different between the sexes were diverse, yet potentially important. The proenkephalin gene *Penk*, which encodes the precursor protein giving rise to enkaphalins, was significantly lower in both the stellate and SCG in female rats. The enkephalins are expressed by a subset of sympathetic neurons [48, 49] and can be co-released with NE in the heart, where they decrease heart rate and blood pressure[50]. In contrast, *Egr1* mRNA was higher in stellate ganglia of female rats. Egr1 is a transcription factor that stimulates expression of the NE biosynthetic enzymes TH and DBH[51, 52]. Egr1 expression is influenced by catecholamines and sex hormones [53, 54], raising the possibility that estrogen could account for the sex difference in stellate *Egr1* expression. Although we did not measure estrogen levels, it is unlikely that hormonal signaling can account for the stellate-specific differences in *Egr1*, *Hpca*, and *Htr2b* mRNA, which are expressed at similar levels in male and female SCG. Target-induced signaling is more likely to explain selective differential expression, and expression of the serotonergic receptors *Htr2b* and *Htr3b* may be related to sex differences in innervation associated with nursing[55]. It is unclear if the gene expression differences detected in this study translate to a difference in the baseline electrophysiology of sympathetic nerves between male and female rats, which may be an important area for future investigation.

Our male-female comparison data in WKY rat ganglia complement other investigations of stellate transcriptomes. A recent study by Bardsley and colleagues compared the stellate ganglion transcriptome of male Wistar and SHR rats [8, 9]. That work identified increased expression of multiple genes in male SHR ganglia that might contribute to excess sympathetic transmission and hypertension [7, 10]. It will be interesting to see if the same genes are dysregulated in female SHR ganglia, and if cardiac NE is higher in the female heart. The most highly expressed stellate ganglion genes in the current study match the most abundant genes in the Bardsley study [8, 9] and our earlier mouse study[2]_,_ providing confirmation of the core stellate ganglion transcriptome. Although we identified sex-specific differences in the expression of many genes, those associated with noradrenergic transmission were expressed at similar levels in male and female ganglia. The identification of differences in tissue neurotransmitter content highlights the need to include both sexes in experiments, and to carefully characterize target tissues as well as the ganglia that innervated them.

## Supporting information

S1 Fig Expression of sympathetic markers. Right stellate ganglion expression levels of genes in both male and female rats using normalized Log10CPM values (Counts Per Million); Mean ± SEM (n = 12). Select genes important in sympathetic function are presented in addition to 3 commonly used housekeeping genes (*Actb*, *Tubb3* and *Hprt1*) for reference. Genes specific to sympathetic neurons are among the most highly expressed in the stellate.
(PDF)

S2 Fig Actin and Tubulin gene isoform expression in the stellate. Average expression across all samples ±SEM (n = 12) expressed as Log10 Counts per Million (CPM).
(PDF)

S3 Fig Synaptotagmin gene isoform expression in the stellate. Average expression across all samples ±SEM (n = 12) expressed as Log10 Counts per Million (CPM).
(PDF)

S4 Fig Syntaxin gene isoform expression in the stellate. Average expression across all samples ±SEM (n = 12) expressed as Log10 Counts per Million (CPM).
(PDF)

S5 Fig Rab3 gene isoform expression in the stellate. Average expression across all samples ±SEM (n = 12) expressed as Log10 Counts per Million (CPM).
(PDF)

S6 Fig Neurotransmitter receptor gene isoform expression in the stellate. Average expression across all samples ±SEM (n = 12) expressed as Log10 Counts per Million (CPM).
(PDF)

S7 Fig Sodium channel gene isoform expression in the stellate. Average expression across all samples ±SEM (n = 12) expressed as Log10 Counts per Million (CPM).
(PDF)

S8 Fig Potassium channel gene isoform expression in the stellate. Average expression across all samples ±SEM (n = 12) expressed as Log10 Counts per Million (CPM).
(PDF)

S9 Fig Western blots for TH and GAPDH in the base of the left ventricle. Equal amounts of protein from 6M and 6F hearts were separated on 4–12% gels and blotted for TH and then GAPDH.
(PDF)

S10 Fig Western blots for TH and GAPDH in the apex of the left ventricle. Equal amounts of protein from 6M and 6F hearts were separated on 4–12% gels and blotted for TH and then GAPDH.
(PDF)

S1 Table Top 100 F v M. The 100 most highly expressed genes in both sexes listed side by side for comparison.
(XLSX)

S2 Table WKY rat expression data. Contains gene identities, raw count expression data, normalized CPM expression data, and statistics for comparing male and female rats. log2FC is the fold change of gene expression in log2 scale (fold difference) male vs. female rats. For example, log2FC of 1 corresponds to fold difference of 2 (up-regulated) vs. female and log2FC of -1 corresponds to fold difference of 0.5 (down-regulated) vs. female. P-value is the empirical Bayes moderated p-value for that particular contrast. FDR is the false discovery rate adjusted p-value.
(XLSX)

S3 Table Gene class analysis. The data from Sheet 1 that were compiled to generate S1–S8 Figs.
(XLSX)
