# Supplementary material for: Sex differences in sympathetic gene expression and cardiac neurochemistry in Wistar Kyoto rats
Source: PLoS One. 2019 Jun 13;14(6):e0218133. doi: 10.1371/journal.pone.0218133 (PMC6564003; doi:10.1371/journal.pone.0218133)
Supplement: S1 Fig — Right stellate ganglion expression levels of genes in both male and female rats using normalized Log10CPM values (Counts Per Million); Mean ± SEM (n = 12). Select genes important in sympathetic function are presented in addition to 3 commonly used housekeeping genes (Actb, Tubb3 and Hprt1) for reference. Genes specific to sympathetic neurons are among the most highly expressed in the stellate. (PDF) [file pone.0218133.s001.pdf]

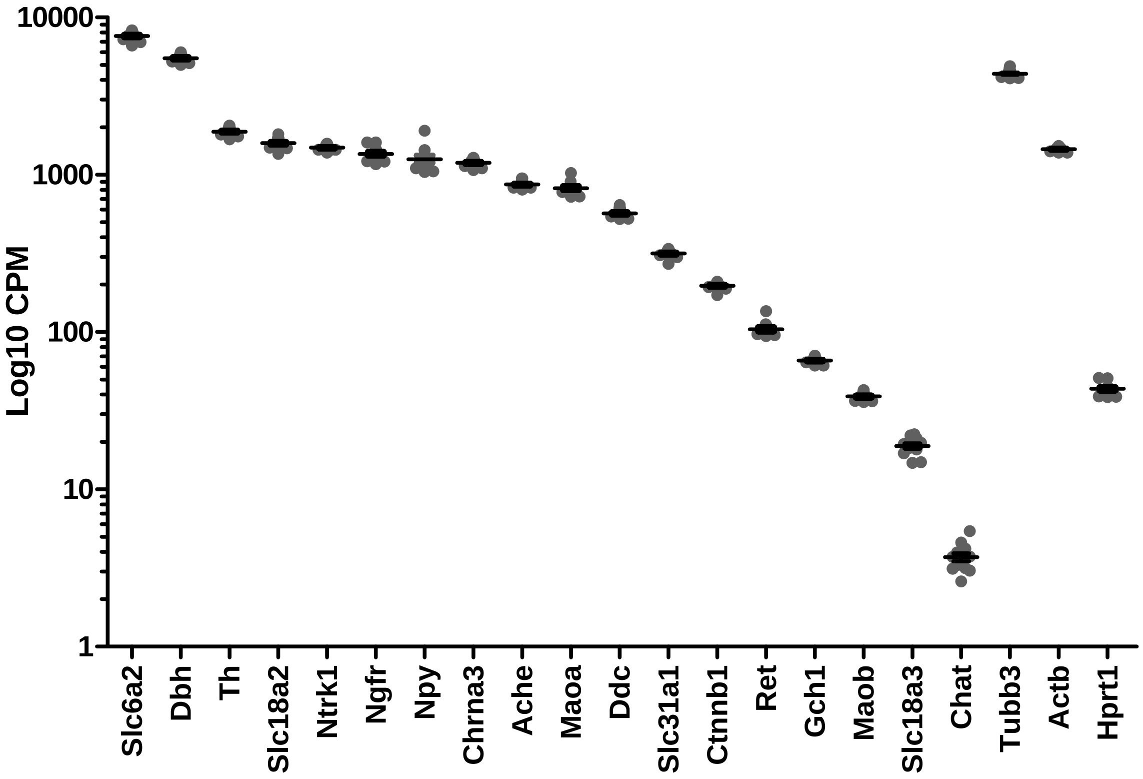

Figure S1: Expression of sympathetic markers

Right stellate ganglion expression levels of genes in both male and female rats using normalized Log10CPM values (Counts Per Million); Mean ± SEM (n=12). Select genes important in sympathetic function are presented in addition to 3 commonly used housekeeping genes (*Actb*, *Tubb3* and *Hprt1*) for reference. Genes specific to sympathetic neurons are among the most highly expressed in the stellate.
