# Supplementary material for: Sex differences in sympathetic gene expression and cardiac neurochemistry in Wistar Kyoto rats
Source: PLoS One. 2019 Jun 13;14(6):e0218133. doi: 10.1371/journal.pone.0218133 (PMC6564003; doi:10.1371/journal.pone.0218133)
Supplement: S8 Fig — Average expression across all samples ±SEM (n = 12) expressed as Log10 Counts per Million (CPM). (PDF) [file pone.0218133.s008.pdf]

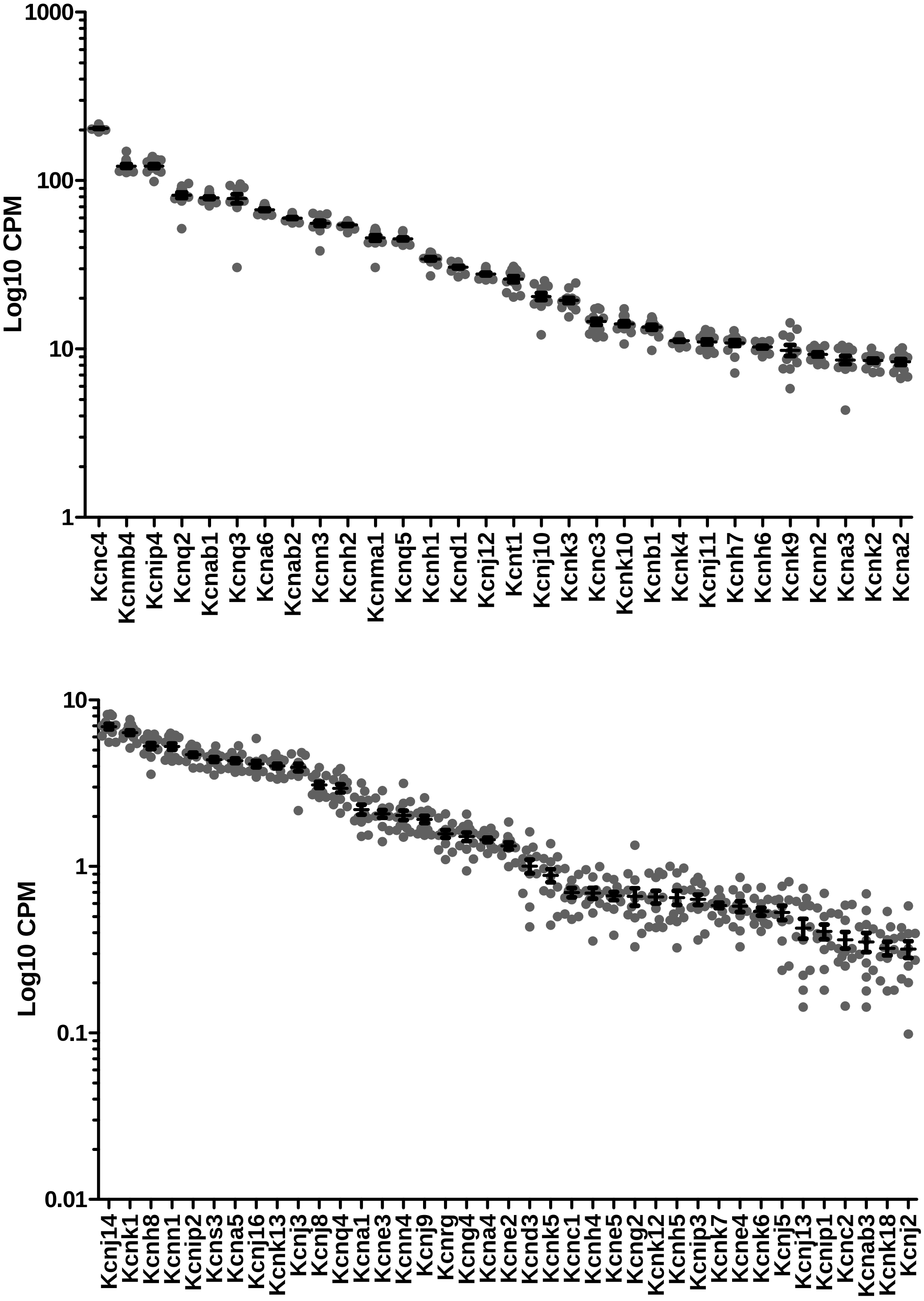

Figure S8: Potassium channel gene isoform expression in the stellate

Average expression across all samples ±SEM (n=12) expressed as Log10 Counts per Million (CPM).
