# Supplementary material for: Sex differences in sympathetic gene expression and cardiac neurochemistry in Wistar Kyoto rats
Source: PLoS One. 2019 Jun 13;14(6):e0218133. doi: 10.1371/journal.pone.0218133 (PMC6564003; doi:10.1371/journal.pone.0218133)
Supplement: S9 Fig — Equal amounts of protein from 6M and 6F hearts were separated on 4–12% gels and blotted for TH and then GAPDH. (PDF) [file pone.0218133.s009.pdf]

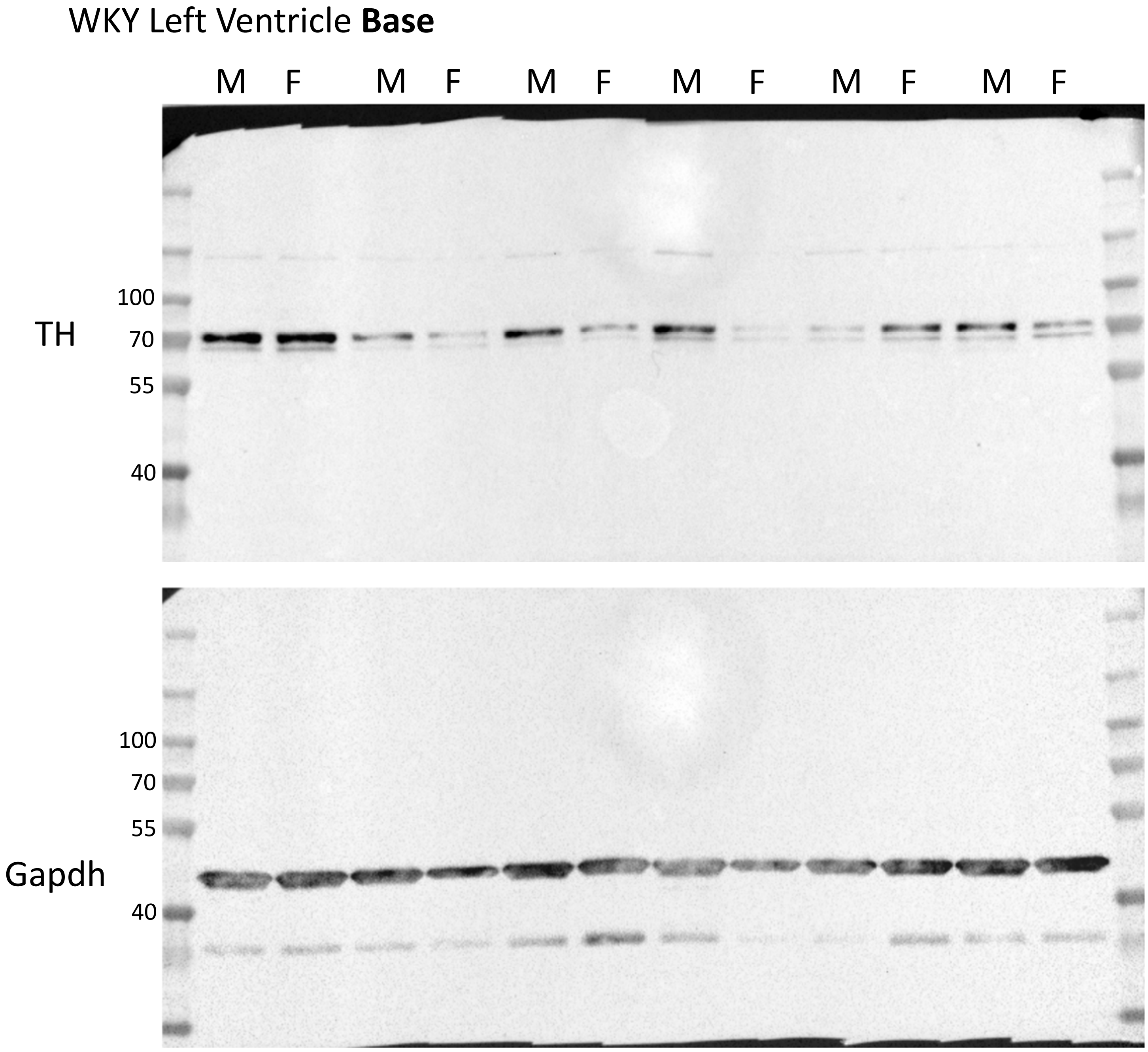

Figure S9: Western blots for TH and GAPDH in the base of the left ventricle.
Equal amounts of protein from 6M and 6F hearts were separated on 4-12% gels and blotted for TH and then GAPDH.
